# Vestibular seizures and spontaneous downbeat nystagmus of ganglioglioma origin: a case report

**DOI:** 10.1186/s12883-023-03311-6

**Published:** 2023-07-24

**Authors:** Ruizhe Yang, Haiyan Wu, Zhiqiang Gao

**Affiliations:** Department of Otorhinolaryngology - Head and Neck Surgery, Peking Union Medical College Hospital, Chinese Academy of Medical Sciences and Peking Union Medical College, Beijing, 100730 PR China

**Keywords:** Vestibular seizures, Downbeat nystagmus, Gangliogliomas, 24-hour Electroencephalogram, Case report

## Abstract

**Background:**

Low-grade gangliogliomas (GGs) are typically epileptogenic intracranial neoplasms. Yet, the presentation of simplex vertiginous experience and spontaneous downbeat nystagmus (DBN) has not been reported to date.

**Case presentation:**

We present the case of a 26-year-old male with focal onset impaired awareness seizures, characterized by vertigo due to right temporal lobe epilepsy caused by ganglioglioma. As rare presentations, a spontaneous, consistent DBN in the absence of vertiginous experience was noticed. MRI suggested lesion in the right temporal pole. Twenty-four-hour continuous electroencephalogram (EEG) monitoring recorded periodic sharp and slow waves, originating from the right temporal lobe. The patient was completely relieved of the symptoms after surgical removal of the tumor, which was histologically confirmed as Grade I Ganglioglioma.

**Conclusions:**

Asides from the cortical pathogenesis of epileptic vertigo, this case also provides insight into the DBN secondary to tumor of the temporal lobe. Moreover, the 24-h EEG is advantageous to recognize vestibular seizures and localize the ictal onset areas.

**Supplementary Information:**

The online version contains supplementary material available at 10.1186/s12883-023-03311-6.

## Background

Vestibular epilepsy is defined as recurrent simple complex or partial seizures that cause vertigo as the predominant symptom [1]. Based on clinical observation, vertigo attacks seldom present as the sole clinical manifestation of epilepsy. Epileptic nystagmus, as an infrequent accompaniment, has been reported in almost all of the patients to be contraversive [2]. Only very few cases of ipsiversive, upbeat and pendular nystagmus have been described [1].

Gangliogliomas (GGs) are rare (1%) intracranial neoplasms, frequently identified with BRAF-6000E mutations (18–30%). Though reported throughout the central nervous system, GGs are typically found within the temporal lobe of children and young adults, as benign, low-grade tumors. GGs are amongst the most common histopathological diagnoses in epilepsy surgical series, accounting for 10% of the cases [3]. Yet, evidence for ganglioglioma in the temporal lobe as etiology of pure vestibular epilepsy has been rare, with scarce case reports of tumors located in internal auditory canal, cerebellopontine angle, cerebellum, or pineal gland, while all in company with symptoms like headache, (multi)sensory loss etc. [4–6]. To our knowledge, this is the first documented case of low-grade intracranial ganglioglioma presenting with isolated vertigo, with a unique sign of spontaneous downbeat nystagmus (DBN).

## Case report

The patient, a 26-year-old right-handed man (financial analyst), was referred to the otolaryngologist complaining about recurrent rotatory vertigo followed by nausea and palpitation. The repetitive experience occurred three to four times a day without any circadian rhythm. The initial attack lasted for about 10 min, while the subsequent attacks were shortened to 10 to 15 s. Onset of the seizure was not related to changes in head position.

**Fig. 1:**
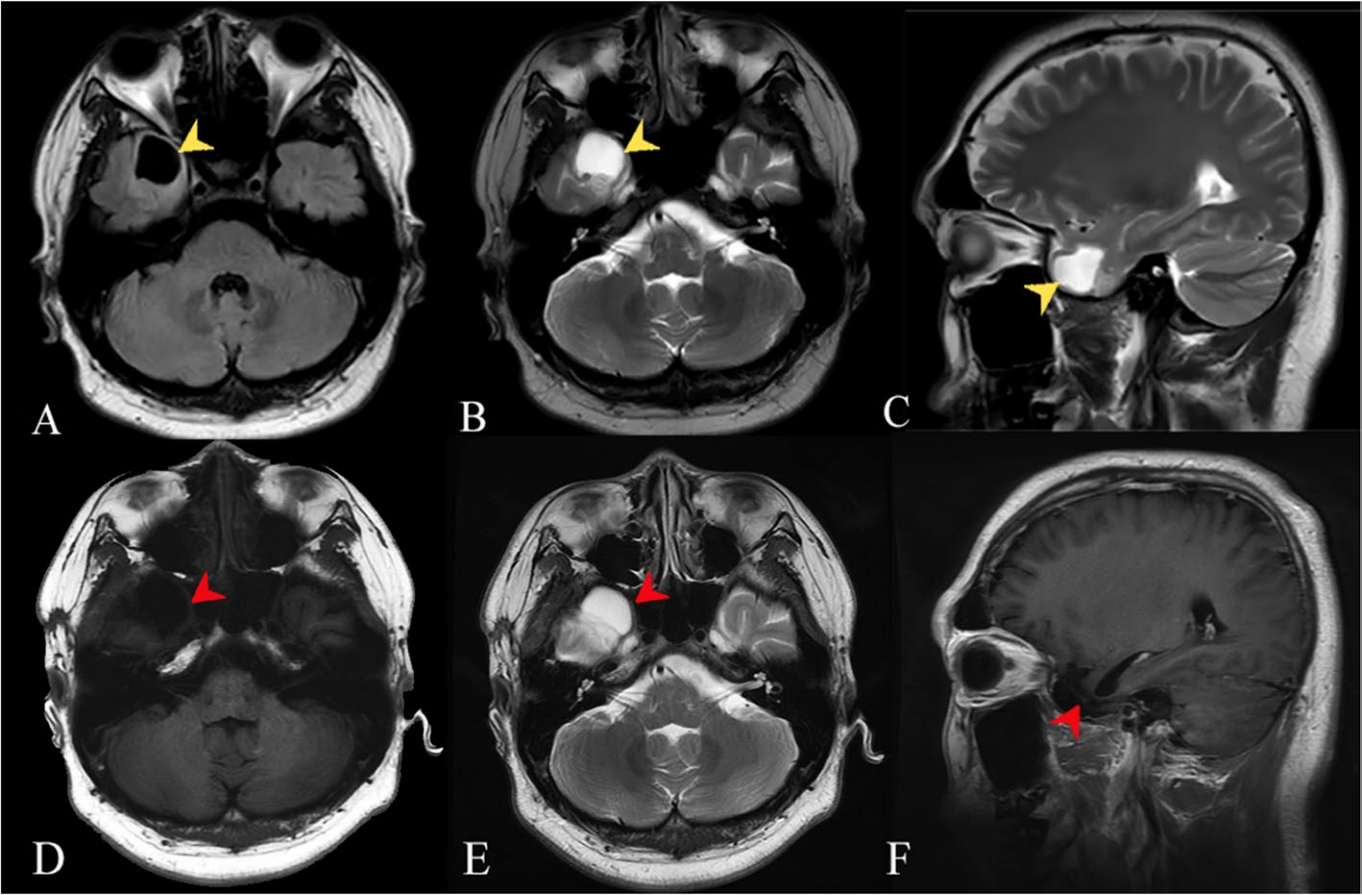
**A**, **B**, **C** The patient’s pre-operative MRI suggested lesion in the right temporal pole with hyperintensity on both T1- and T2-weighted image. **C**, **D**, **E** Follow-up MR images performed six months after the surgery showed increased T1 and T2 signal intensity but no focal enhancing lesion in the right temporal lobe, suggesting post-operative change without residual tumor

Before this visit, these symptoms had repetitive for half a year, responsive to neither physical therapy nor Lorazepam. Orthopedist had ruled out the cervical origin by finding an almost natural curve of the cervical spine. Although brain magnetic resonance (MR) scanning revealed an irregularly shaped lesion in the right temporal lobe, measuring 2.0 × 2.4 cm (Fig. 1), no causality was established due to negative electroencephalogram (EEG).

**Fig. 2:**
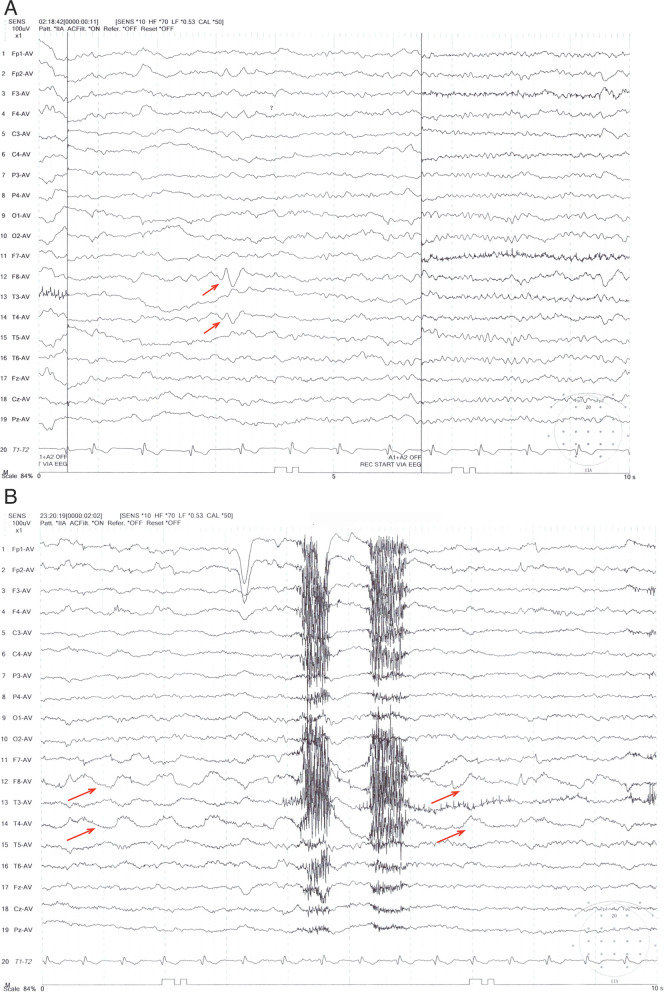
**A** This EEG was recorded in the deep sleep state. Note the sharp wave followed by slow wave at the right anterior (F8) and mid-temporal leads (T4). **B** The slow-wave components of the interictal epileptiform discharges arising from the right temporal region, consistent with location of the lesion

At our clinic, we noticed a consistent, spontaneous DBN (Video 1), which intensified by head shaking test, while diminished at supine position. Results of caloric test and audiometry were both unremarkable. However, during the follow-up appointment, the patient reported an on-site brief attack with déjà vu experience, highly suggestive of further neurological investigations. Hence, a 24 h-EEG monitoring was additionally performed where ictal sharp and slow waves of temporal lobe origin (Fig. 2a and b), in accordance with onsets of vertigo and concomitant palpitation, were recorded. Rhythmic changes lasting 15 to 20 s detected by synchronous EEG agreed with this finding. Hence, diagnosis of vestibular epilepsy was established.

**Fig. 3:**
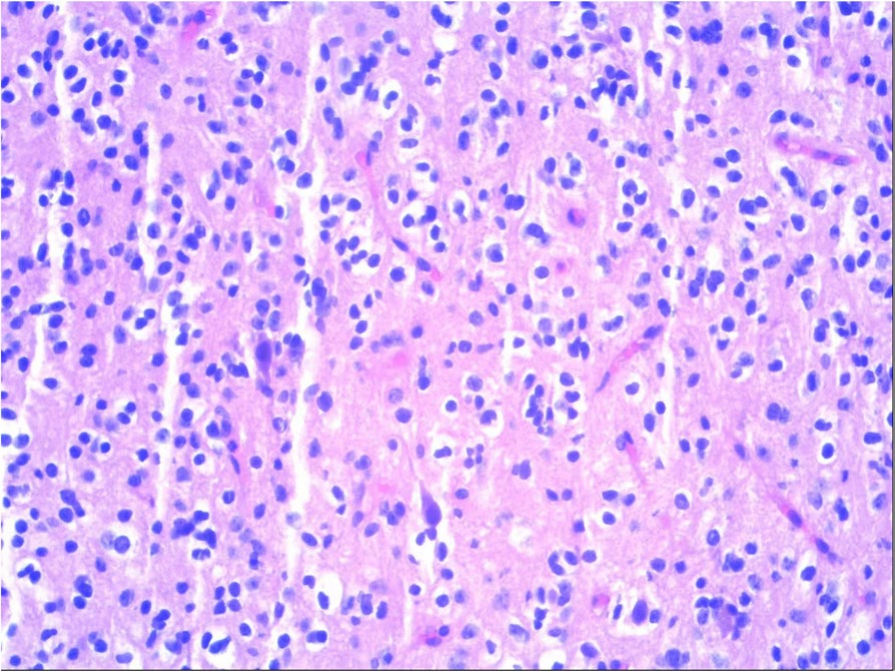
Ganglion cells contained Nissl substance and vesicular nuclei with prominent nucleoli and were randomly distributed in a haphazard fashion without polarization of cell processes

After only four days of administration of Oxcarbazepine (300 mg bid), the onset frequency reduced to once per day or less while duration shortened into 2 s. At Day 10 of the treatment, DBN became feeble, yet remained detectable (Video 2). Despite the efficacy of anti-convulsant, the patient demanded surgical removal of the tumor. The lesion of the right temporal lobe was resected en bloc. Although hyperintensity on both T1- and T2-weighted MR images were observed six months after the procedure, no sign of nodal or peripheral enhancement suggest complete removal of the lesion without recurrence. Histopathologic analysis of the tumor demonstrated classical grade I findings: circumscribed neuroglial tissue composed of mature ganglion cells existing in a background of low-grade astrocyte-like cells with coarse processes (Fig. 3H&E ×200). Ganglion cells contained Nissl substance and vesicular nuclei with prominent nucleoli and were randomly distributed in a haphazard fashion without polarization of cell processes. Molecular-genetic diagnosis included BRAF-V600E mutation. Post-operatively, the patient continued treatment with Oxcarbazepine (300 mg bid) for six month, during which period only three mild attacks took place. At the time of publication, the patient has remained symptomless for the past 19 months.

## Discussion

Although epilepsy are the most common presenting signs of GG, this tumor has rarely been reported as cause for vestibular seizures. This case, to our knowledge, is the first documented temporal-based GG resulting in epileptic vertigo coupled with spontaneous DBN.

The episodic vestibular presentations can be attributed to focal discharge of vestibular cortex secondary to the neoplasm. Increased cortical excitability due to edematous peritumoral brain tissue, elevated intracranial pressure, metabolic derangement etc., might account for the tumor-associated epileptogenicity [7]. Such space-occupying lesions are predominantly localized in the temporo-parietal junction (TPJ), known as the vestibulogenic area. Moreover, tumor-associated seizures are generally marked with progression in parallel with the tumor growth, anti-convulsant drug-resistance, and increased duration and severity. This patient, however, depicted his symptoms as stereotyped, and monotonous vertigo in isolation, which is responsive to Oxcarbazepine.

Unusual findings also include the spontaneous DBN, whilst epileptic nystagmus is predominantly horizontal or, less commonly, upbeat [2]. Two characteristic manifestations indicated that DBN in this patient was more attributable to intracranial hypertension (ICH) than to seizures. Firstly, the nystagmus gradually intensified while achieving sitting position, and diminished when shifting to supine position. Positional dependence suggested a link between DBN and pressure change of cerebrospinal fluid (CSF), which have been reported worldwide. Although DBN in this case presented as the only sign of ICH since the tumor grew at a slow pace, the radiological findings of a slightly enlarged ipsilateral hippocampus shifting to the right cerebral peduncle lent support to this hypothesis. A limitation of this study is not having included the CSF pressure at different positions as the evaluation indicators.

Secondly, after epileptic attacks were resolved by Oxcarbazepine on Day 10, DBN turned feeble but remained detectable via video-goggles. Oxcarbazepine blocked the voltage-gated sodium channel to inhibit repetitive neuronal firing and spreading [8]. Meanwhile, Oxcarbazepine is also associated with an elevated gamma aminobutyric acid (GABA) activity in the central region [9]. GABA functioned as a principal inhibitory neurotransmitter in Purkinje fibers and their downstream effector cells. It has been acknowledged that DBN arise from activated anterior canal pathways, which is normally inhibited by cerebellar flocculus [10]. Thus, disinhibition to flocculus can produce downbeat nystagmus. The fact that DBN in our patient was considerably alleviated by Oxcarbazepine, suggested potential existence of GABAergic transmission from Purkinje cells to floccular neurons [11]. In this case, the inhibitory control of Purkinje cells in cerebellum over anterior projections was impaired due to chronic ICH secondary to the tumor growth. The GABAergic neurons transmitted weakened the inhibitory signals from the injured Purkinje cells to flocculus, which generated DBN. Administration of Oxcarbazepine enhanced GABA receptor activity, bringing down the excitability of anterior projections, and therefore, relieving the DBN [12]. Similar pathogenesis has been discussed by Wang [13] and van Rootselaar [14] in Familial Cortical Myoclonic Tremor with Epilepsy, where cerebellar grey matter loss underlie cortical tremors with DBN.

Finally, this report highlighted the importance of 24 h-EEG, without which the temporal lobe epileptic discharge might have escaped detection. Clusters of onsets, subjective description of déjà vu, and intracranial structural abnormality were evidence that should invite the clinicians to associate the symptoms and signs with seizures. Regardless of the rarity of intracranial lesions as causes of vestibular epilepsy, it should be included in the differential diagnosis of isolated, recurrent, spontaneous vertigo. To establish the diagnosis, thorough investigations should involve not only the examinations depending on real-time recording, but also the ambulatory EEG that enables 24-h continuous monitoring of the electrical activity.

## Conclusion(s)

In conclusion, cerebral tumors can be rare etiology of isolated vertigo. Primary vertical nystagmus suggests central vestibular disorders, and déjà vu experience indicates potential lesions in the temporal lobe. Twenty-four-hour continuous EEG monitoring provided essential evidence for identification of the causative lesion, which have been occasionally missed by other traditional investigations.

## Supplementary Information

**Additional file 1.**

**Additional file 2.**

## Data Availability

Not applicable.

## Abbreviations

GGs: Gangliogliomas
EEG: Electroencephalogram
DBN: Downbeat nystagmus
MRI: Magnetic resonance imaging
TPJ: Temporo-parietal junction
CSF: Cerebrospinal fluid
